# Reassessing Biological Threats: Implications for Cooperative Mitigation Strategies

**DOI:** 10.3389/fpubh.2015.00251

**Published:** 2015-11-30

**Authors:** Summer Elise Galloway, Stephanie Rachel Petzing, Catharine Grace Young

**Affiliations:** ^1^Department of Defense, American Association for the Advancement of Science and Technology, Washington, DC, USA

**Keywords:** biological threats, mitigation strategies, Global Health Security Agenda, One Health, Cooperative Threat Reduction Program, Department of Defense, antimicrobial resistance, biosurveillance

## Abstract

Multiple factors ranging from globalization to ecosystem disruption are presenting the global community with evolving biological threats to local, national, and global security that reach beyond the realm of traditional bioweapon threats. As a result, mitigation strategies have adapted necessarily to the increased diversity of biological threats. In general, response and preparedness strategies have largely shifted from being primarily reactive to traditional biological weapons to more proactive in nature. In this review, we briefly explore biological threats through a wider aperture, to embrace a greater appreciation of viral pathogens, antimicrobial resistance, and agricultural pathogens, and their potential to cause civil, economic, and political devastation. In addition, we discuss current mitigation strategies codified by the Global Health Security Agenda and the One Health paradigm as well as some of the available tools to assist with their sustainable implementation.

## Introduction

The world has entered a new era of biological threats due to unprecedented changes brought by globalization, growing agricultural demands, the diffusion of advanced biotechnologies, and insufficient reporting of outbreaks. Outbreaks of zoonotic diseases for which adequate treatments or vaccinations are unavailable or of diseases that could cripple the agriculture sector are examples of “non-traditional” biological threats with the potential to cause public health and economic devastation. Although these threats fall outside the traditional boundaries of bioterrorism, they have become a major target of the biodefense community in order to protect U.S. Armed Forces and citizens at home and abroad as well as our allies. Mitigating these threats requires the close cooperation of the national security, public health, and agriculture communities within the United States, partner nations, international organizations, and non-government organizations.

This article provides an overview of the non-traditional biological threats posed by emerging viruses, antibiotic-resistant bacteria, and agricultural pathogens. The article then describes efforts by the government sector [e.g., the Global Health Security Agenda (GHSA)] and the non-government sector (e.g., the One Health paradigm) to develop frameworks to address these threats. Finally, the article offers an assessment of the contribution that the Cooperative Biological Engagement Program (CBEP) within the Department of Defense’s Cooperative Threat Reduction (CTR) program has made to global health security through its programs to strengthen biosurveillance (BSV) and building partner capacity through collaborative research.

## Viral Threats

While viruses are considered traditional biological threats by the defense and security sectors, they are typically discussed in terms of bioengineering to increase pathogenicity or “weaponize” rather than appreciating the threat they pose naturally. In this regard, an understanding of the health care system, cultural practices of the affected population, and political and economic climate of the affected region are critical and often underestimated aspects of dealing with viral outbreaks. Even when these factors are understood, the capabilities of governments and non-government organizations to respond effectively are complex and legitimate.

There are a number of viral threats to public health as well as regional, national, and global health security. However, the overwhelming majority of viruses on the Centers for Disease Control and Prevention and the United States Department of Agriculture “Select Agents” lists are RNA viruses, which are among the most serious uncontrolled causes of extant and emerging infectious diseases (1, 2). Owing to the nature of their replication machinery, which lacks proofreading activity and therefore has high error rates, RNA viruses exist as quasi-species or a “molecular swarm” of viral genomes. This leads to each having different levels of fitness within the population and positioned to respond readily to selective pressure (3–5). This feature affords RNA virus populations the capability to evolve at rates of up to one million times faster than their DNA counterparts, presenting a daunting public health challenge when designing therapeutics and vaccines. This is exemplified by the antiviral resistance in HIV and influenza virus populations, as well as the evolution of influenza A virus, which necessitates the annual reformulation of the vaccine. Other more genetically stable viruses, such as the DNA virus causing smallpox, have also been considered traditional biological threats. However, due to the availability of an effective vaccine, the success of the eradication campaigns over the last 45 years, and the consolidation of virus stocks, smallpox is generally disregarded as a public health threat. It has been speculated that due to the cessation of mass vaccination in 1980 and waning immunity, less than 20% of the population is sufficiently protected from infection (6). As such, the result of an outbreak of smallpox, either accidental or intentional, could be devastating to a regional, national, or global population.

The recent Ebola virus (an RNA virus that causes hemorrhagic fever) epidemic in West Africa highlights the civil, economic, and public health system destabilization that can occur as the result of a naturally occurring epidemic, let alone an intentional outbreak. For example, the epidemic has resulted in over 27,000 reported cases and more than 11,000 deaths, with an economic impact on Guinea, Sierra Leone, and Liberia rising to approximately 5% of their combined GDP in 2014 and 12% in 2015 (7). This outbreak, along with the potential for other similar catastrophic events, illustrates that having effective global BSV capabilities and response plans in place are crucial in mitigating viral pathogen threats.

## Antimicrobial Resistance

Antimicrobial resistance is appreciated in the public health arena as an emerging threat, but is only beginning to be explored in the biological defense sector. The threat of especially dangerous pathogens, which have either naturally or through genetic engineering acquired resistance to vaccines or antimicrobials, is severe (8). Genes conferring resistance can be carried on mobile genetic elements (e.g., plasmids), which can be inconspicuously maintained in less concerning bacterial species and transferred through natural mechanisms to pathogenic agents (9). This is illustrated by a natural plague outbreak in Madagascar (1995), where a patient was infected with a plague isolate that was resistant to streptomycin, chloramphenicol, tetracycline, sulfonamides, ampicillin, kanamycin, and spectinomycin, all of which are first- and second-line drugs used to treat and prevent plague (10). Further investigation revealed that the genes for this alarming pattern of resistance were carried on a plasmid originating in the Enterobacteriaceae family, which was able to be transferred from *Escherichia coli* to the plague bacterium. This demonstrates the real possibility of dangerous pathogens obtaining antimicrobial resistance genes from innocuous bacteria in the environment. This evolving threat has received highest attention with the issuance of Executive Order 13676: “Combating Antibiotic-Resistant Bacteria” by President Barack Obama in September 2014 (11) and a corresponding “National Action Plan for Combating Antibiotic-resistant Bacteria” released in March 2015 (12).

## Agricultural Pathogens

Despite the agriculture sector playing a formidable role in the economic, social, and political stability of the U.S., it has yet to receive the needed attention with respect to protection against major biological threats. Unlike the developing world, if a devastating outbreak of either animal or plant disease occurred in the U.S. (e.g., African swine fever, soybean rust, and potato wart), it would be the severe economic consequences that would pose as the greatest threat, not famine (13). A powerful example is the case of foot-and-mouth disease (FMD) outbreak in the United Kingdom (2001), a country previously free of the disease. FMD is a viral disease that affects cloven-hoofed animals, and while it is a disease of low mortality, it is highly infectious and spreads via aerosols or direct contact with contaminated equipment or feed. Following the first outbreak, the European Union blocked imports of British beef, sheep, and swine, leading to an unprecedented loss of revenue of approximately $13 billion and the culling of approximately 10 million cattle and sheep. For the U.S., annual sales of beef are in the realm of $40 billion, illustrating that the trade consequences of an FMD outbreak could be just as devastating. Surprisingly, the impact in countries deemed FMD-free has received more attention than the impact of outbreaks in FMD-endemic countries, despite the annual impact of FMD-related losses, estimated to be up to $21 billion. As with public health threats, mitigation of agriculture threats face many of the same obstacles and requires a well-coordinated, timely, and robust animal disease-reporting system. In addition, worldwide control of agricultural pathogens requires coordination within and between countries requiring both national and international public investment.

It is clear from the global spread and economic destruction of infectious diseases such as Ebola and FMD and the emergence of antimicrobial resistance pathogens that a new approach to combat global biological threats is required. Increased globalization of trade and travel has resulted in an international community that is not secure from biological threats. Importantly, more than 80% of countries did not meet the World Health Organization deadline for being prepared for infectious disease threats. Severe Acute Respiratory Syndrome (SARS) in 2003 is a primary example of this. In under a year, SARS swept 29 countries, was diagnosed in over 8000 patients, and led to 774 deaths (14). The rapid spread of SARS was made possible by the unprecedented volume and speed of international travel and the inability of countries to handle the detection, diagnosis, and reporting of the disease.

## Global Health Security Agenda

To tackle such issues, the GHSA, a new multisectoral and interagency government approach to combat global threats, was launched by the U.S. and international partners in 2014. The major objectives of the GHSA are to prevent avoidable epidemics, detect threats early, and respond rapidly and effectively on a global scale. Incidents such as the SARS outbreak highlight that one nation cannot achieve global health security on its own. As such, the U.S. has committed to working with 30 countries over the next 5 years to implement the GHSA. This strategic approach is aimed to specifically strengthen disease response capabilities and rapidly detect and improve transparency in outbreak reporting by supporting existing agreements under the World Health Organization International Health Regulations 2005 (15), the World Organization for Animal Health Codes (16), and the Codex Alimentarius International Food Standards (17). In short, the GHSA is a collaborative effort to secure the world from global health threats posed by infectious diseases, whether naturally occurring, or the result of an accidental or intentional release of pathogens.

## One Health

Estimates indicate that approximately 75% of emerging or reemerging infections are vector-borne or zoonotic. Within the last 20 years, there have been several major instances of cross-species transmission that have caused severe public health, economic, and political consequences, not to mention effects on public confidence in the ability of governments to respond to emerging biological threats. Examples include the transmission of H5N1 to humans that was first reported in 1997 (18, 19); the West Nile virus outbreak in 1999 that possibly originated from illicit animal importation into New York (20); the SARS coronavirus (CoV) epidemic in 2003 originating from bats and/or civets (21, 22); the H1N1 influenza A virus pandemic of 2009 originating from swine (23); the Middle East Respiratory Syndrome CoV epidemic in 2012 that likely originated from bats and/or camels (24, 25); the transmission of H7N9 IAV to humans in 2013 from poultry (26); and the most recent Ebola virus epidemic in 2014 that was likely transmitted to humans by bats. These examples underscore how devastating zoonotic diseases can be, even if rapidly, detected and geographically contained.

The concept of “One Health” is not new, as practitioners have long recognized the connection between animal and human disease, but it has achieved greater traction among the human and animal public health sectors in recent years. The American Veterinary Medical Association defines One Health, in broad terms, as “the collaborative effort of multiple disciplines – working locally, nationally, and globally – to attain optimal health for people, animals and the environment.”(27). Achieving this goal is articulated by the One Health Initiative that includes several joint efforts in: (1) the education of stakeholders; (2) communication in journals, conferences, and health networks; (3) clinical care through assessment, treatment, and prevention of cross-species transmission; (4) implementing disease surveillance systems; (5) achieving a better understanding of factors contributing to zoonotic transmission; (6) the development and evaluation of new diagnostics, therapeutics, and vaccines; and (7) the education of leaders and the public sector through responsible journalism (28).

There are undeniable benefits to operating under the One Health umbrella, but consideration of unintentional consequences is worth noting. For example, how should the economic implications of reporting outbreaks in animal populations balance against public health? Are there mechanisms in place to reduce the severity of disruptions to a fledgling economy to bolster reporting levels? Regardless of the barriers, what is clear is that achieving the vision of the One Health Initiative will require substantial and sustained international commitment in terms of funding, research, public and animal health capacity building, and infrastructure development.

## The Cooperative Threat Reduction Program

The CTR Program has evolved from the original Nunn-Lugar CTR program, which focused on materials and expertise left behind by the Soviet regime, into a comprehensive program to reduce worldwide threats from nuclear, radiological, chemical, and biological weapons. A central tenet of the CTR Program is sustainability, as the ultimate vision is that partner countries have the appropriate facilities that incorporate safety and security standards, necessary training, and technical expertise to effectively mitigate nuclear, chemical, and biological threats within their borders.

Within CTR, CBEP engages with partner country governments, institutions, and scientists to reduce the threat from traditional biowarfare agents, as well as agents that do not have a history of attempted weaponization, but are of security concern due to their potential to cause mortality, economic, or civil disruption. As the nature of biological threats evolves, CBEP has demonstrated the flexibility and agility to address the challenge, by encouraging the safe and secure development of biological research and scientific workforce capacity. Below, we focus on two major mechanisms for accomplishing the CTR mission: biosurveillance, which inherently incorporates biosafety and biosecurity elements, and the Cooperative Biological Research (CBR) program within CBEP.

## Biosurveillance

Biosurveillance refers to the continual process of gathering, analyzing, and interpreting data in order to achieve early detection, warning, and awareness of biological threats to human or animal health as well as to national, regional, and global security. BSV capability requires trained epidemiologists and clinicians, laboratory capability to diagnose disease, and information systems to manage and relay disease information to decision makers responsible for managing outbreak responses. The foundation of a functioning BSV system is a capable network of local laboratories that serve as spokes to a central reference laboratory for diagnostic and reporting purposes. Together with host governments, the U.S. interagency, and non-government organizations, CBEP improves capabilities that support sustainable and integrated laboratory networks in partner countries. Notable benefits of engaging partner countries on BSV-related efforts are the overall safety and security culture that may be imparted on professionals in the BSV network and by investing in coordinated surveillance of human, animal, and plant health, not only does overall health improve, but practitioners have an increased ability to detect outbreaks of pathogens of security concern, which may lead to more timely implementation of mitigation strategies.

Cooperative Biological Engagement Program employs several core strategies in working with a partner country to enhance BSV capacity and capability, including building secure laboratory infrastructure and identifying and training laboratory personnel and clinicians to conduct safe and secure diagnostics, especially when working with dangerous and highly infectious pathogens. CBEP also supports the implementation of BSV-based research to monitor endemic diseases in order to differentiate between natural, accidental, and deliberate outbreaks, as well as information system networks to manage health-related data, critical to BSV, for the purpose of analysis and reporting to the authorities responsible for implementing specific biological mitigation strategies.

The implementation of a BSV system can be complicated; there is no universal solution, and multiple factors should be considered in order to strike the appropriate balance between specificity and sensitivity. These include the cost of implementation, most effective type of BSV that should be implemented for a given country or disease (active vs. passive and syndromic vs. laboratory surveillance), most effective information and analysis system to use, laboratory capabilities of the country, and extent of known biological markers of disease. The assessment of these factors requires a collaborative and interdisciplinary approach ranging from engagement with public and animal health practitioners to coordination with government officials and law enforcement. Considering the gamut of collaboration, CBEP is uniquely positioned to affect a multitude of capabilities that ultimately lead to improved local, national, and global health security.

## Cooperative Biological Research

As illustrated by the Ebola outbreak in West Africa, a naturally occurring outbreak can quickly become a transcontinental threat, the mitigation of which requires a modern approach. This includes building networks of professionals trained in the safe and secure conduct of biological research and surveillance. CBEP’s CBR funds collaborative projects with researchers from partner countries. The projects include elements of biosafety and biosecurity training, and the research topics are strategic, furthering the interests of the partner countries as well as the objectives of CBEP. These efforts contribute to global BSV of human and animal pathogens endemic in partner countries, providing a baseline prevalence of biological disease threats within a geographic area. Understanding the incidence and natural variations of pathogens in an ecosystem is crucial to the investigation of an unusual outbreak and to the public, animal, and/or plant health response. These data help to determine the source of an outbreak as well as make a rapid determination of whether an increase in infections or antimicrobial resistance is natural or due to an accidental or intentional release. The cooperative nature of the CBR program provides a platform for the international collaborative work necessary to address transboundary threats such as zoonotic diseases carried across borders by infected humans, importation of animals, or natural movement of mobile vectors such as bats. These diseases require a comprehensive One Health approach involving both human and animal health sectors, which CBR facilitates.

Moreover, the scientific collaboration encouraged by the CBR program is an example of the diplomacy and engagement that fosters continued communication networks among the international community that are crucial during outbreaks of pathogens. The language of science is universal, and the value of international collaboration is clear to those engaged in scientific research. Scientists welcome shared information regarding data, protocols, equipment, and training, and interactions of this nature are excellent starting points for U.S. engagement, as well as collaboration among partner countries, facilitating peaceful collaboration and free exchange of information.

## Conclusion

The world is constantly changing: humans across the globe are more interconnected and, due to habitat encroachment and ecological shifts, are in closer and more frequent contact with animal reservoirs of disease than ever before. This combined with free information flow, rapid scientific development, and the ever-present potential of misuse of biological agents presents a new and challenging environment for biological threat reduction requiring an adapted strategic approach. Open, effective, and consistent international collaboration for surveillance of pathogens is a backbone of this new approach. It must be comprehensive and include emerging threat agents, which are not necessarily limited to the traditional bioweapon agents of the Cold War era. Safe, secure, and sustainable capacity development in partner countries to increase the network of trained biological science professionals, as well as interconnected and effective infrastructure, is necessary. CBEP combines international agility and experiences with cutting-edge technical expertise to not only effectively reduce the threat from “traditional” bio-warfare agents but also emerging biological threats.

## Disclaimer

The views presented in this article represent those of the authors and do not necessarily reflect the position of the the Department of Defense.

## Conflict of Interest Statement

The authors declare that the research was conducted in the absence of any commercial or financial relationships that could be construed as a potential conflict of interest.
